# Synthesis, Characterization, and Anticancer Activity of Benzothiazole Aniline Derivatives and Their Platinum (II) Complexes as New Chemotherapy Agents

**DOI:** 10.3390/ph14080832

**Published:** 2021-08-23

**Authors:** Md. Kamrul Islam, Ah-Rum Baek, Bokyung Sung, Byeong-Woo Yang, Garam Choi, Hyun-Jin Park, Yeoun-Hee Kim, Minsup Kim, Seongmin Ha, Gang-Ho Lee, Hee-Kyung Kim, Yongmin Chang

**Affiliations:** 1 Institute of Biomedical Engineering Research, Kyungpook National University, 680, Gukchaebosang-ro, Jung-gu, Daegu 41944, Korea; mkislam2008@yahoo.com; 2 Department of Medical & Biological Engineering, Kyungpook National University, 80, Daehak-ro, Buk-gu, Daegu 41566, Korea; baxun@naver.com (A.-R.B.); xxgzxz4@naver.com (B.S.); byungwoo1128@naver.com (B.-W.Y.); 3 R&D Center, Mirae BioPharm. Co., 124, Sagimakgol-ro, Jungwon-gu, Seongnam-si 13207, Korea; garam1458@naver.com (G.C.); phj0808@naver.com (H.-J.P.); bigeye38@naver.com (Y.-H.K.); 4 InCerebro Drug Discovery Institute, Seoul 01811, Korea; minsupkim.bio@gmail.com; 5 Department of Biomedical Science, School of Medicine, Kyungpook National University, 680, Gukchaebosang-ro, Jung-gu, Daegu 41944, Korea; zx996574@gmail.com; 6 Department of Chemistry, Kyungpook National University, 80, Daehak-ro, Buk-gu, Daegu 41566, Korea; ghlee@knu.ac.kr; 7 Laboratory Animal Center, the Daegu-Gyeongbuk Medical Innovation Foundation, 88 Dongnae-ro, Dong-gu, Daegu 41061, Korea; 8 Department of Molecular Medicine, School of Medicine, Kyungpook National University, 680, Gukchaebosang-ro, Jung-gu, Daegu 41944, Korea; 9 Department of Radiology, Kyungpook National University Hospital, 130 Dongdeok-ro, Jung-gu, Daegu 41944, Korea

**Keywords:** benzothiazole aniline (BTA), platinum (II), anticancer, DNA, liver, docking

## Abstract

We describe the synthesis, characterization, molecular modeling, and in vitro anticancer activity of three benzothiazole aniline (BTA) ligands and their corresponding platinum (II) complexes. We designed the compounds based on the selective antitumor properties of BTA, along with three types of metallic centers, aiming to take advantage of the distinctive and synergistic activity of the complexes to develop anticancer agents. The compounds were characterized using nuclear magnetic resonance spectrometry, Fourier transform infrared spectroscopy, mass spectrometry, elemental analysis, and tested for antiproliferative activity against multiple normal and cancerous cell lines. **L1**, **L2**, and **L1Pt** had better cytotoxicity in the liver, breast, lung, prostate, kidney, and brain cells than clinically used cisplatin. Especially, **L1** and **L1Pt** demonstrated selective inhibitory activities against liver cancer cells. Therefore, these compounds can be a promising alternative to the present chemotherapy drugs.

## 1. Introduction

**Figure 1:**
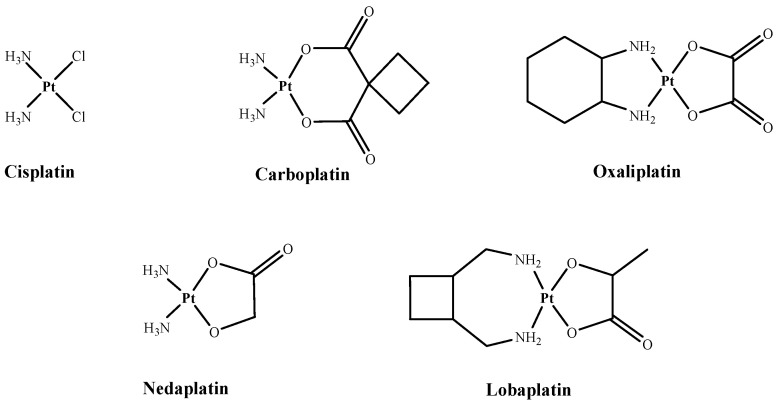
Structure of the clinically approved platinum complexes.

The success of cisplatin [*cis*-diamminedichloroplatinum-(II)] flagged the way for second-and third-generation cancer drugs: carboplatin, oxaliplatin, nedaplatin, and lobaplatin (Figure 1) [1,2,3]. The efficacy of cisplatin is governed by its ability to covalently bind with DNA and change its helical structure, resulting in cell death [4]. However, new anticancer agents have drawn attention for the reason that current platinum agents present drug resistance and have several limitations, such as a lack of selectivity, poor pharmacokinetic profiles and inadequate water solubility [5,6,7,8]. Moreover, existing platinum-based chemotherapy drugs are mostly correlated with general toxicity, which causes adverse side effects [9,10,11,12,13]. An additional change to cisplatin may regulate these toxic effects. For instance, the substitution of the amine group in cisplatin using 1,2-ethylenediamine forms a structure that is used in various structure–activity relationship (SAR) studies, and proper substitution of the ethane bridge by aromatic groups may increase the cytotoxicity [14,15,16,17]. As well, pyridine moiety was also used in several Pt chemotherapeutics, which have shown promising antiproliferative properties [18,19].

Benzothiazole aniline (BTA), which is chemically known as 2-(4-aminophenyl)-benzothiazole, is a fused heterocyclic pharmacophore that exhibits antitumor activity [20]. Substituent exchange of the 3′-position with methyl or halogens (for instance Cl) contributes to the increased antitumor activity against various cancer cell lines (ovarian, colon, renal, etc.) [21,22]. Moreover, BTA showed selective anticancer activity by demonstrating a distinct cytotoxic response against some tumor cell lines, while no hormonal dependency was recognized [23,24]. Therefore, several studies have been performed in the past years to develop novel BTA derivatives as anticancer agents [25,26,27,28]. For example, the ring-substituted BTA derivative 2-(4-amino-3-methylphenyl) benzothiazole—DF 203—was developed as an antitumor agent, and phortress NSC 710,305 underwent phase 1 clinical trials as a prodrug [24,25]. In addition, technium (^99m^Tc) and rhenium (^186^Re, ^188^Re) complexes of BTA conjugate were developed as radiopharmaceuticals for targeted therapy and imaging of breast cancer [29,30]. In our previous study, we developed a bifunctional Gd–DO3A–BTA chelate and evaluated its antiproliferative activity, both in vivo and in vitro, as a theranostic agent [31]. Recently, Mavroidi et al. synthesized Pd (II) and Pt (II) chelates of BTA derivatives to target cancer cells, however, the studied compounds proved less cytotoxic than clinically permitted cisplatin [32]. The approach using different types of biologically active moieties is frequently used in the design of novel metal-based drugs [33,34,35]. For example, a series of biologically active organometallic compounds were synthesized bearing an acetylsalicylic acid (ASA) substructure to inhibit cyclooxygenase (COX) enzymes [36]. Also, the nonsteroidal anti-inflammatory drug was conjugated to Pt (II) with various intracellularly cleavable linkers, which disclosed potent cytotoxic activity against different cancer cell lines [37]. Similarly, few novel Pt (II) complexes were synthesized bearing aliphatic amines and 1,8-naphthalimide moieties with DNA-targeting properties to achieve more potent and selective metallodrugs [38].

Based on the concepts containing biologically active molecules and the cisplatin-like composite, we designed and synthesized novel benzothiazole aniline derivatives (**L1**, **L2**, and **L3**) and their corresponding Pt (II) complexes as selective agents for treating cancer. We have hypothesized that the conjugation of new ligands using BTA will result in compounds that have distinct cytotoxic properties as BTA is well known for its antitumor activity. In vitro screening was performed in different cancer and normal cell lines, and stability of the lead compounds was measured in buffered aqueous solution. In addition, we conducted molecular modeling studies to predict the best binding poses of ligands in the active site of DNA. We hypothesized that **L1** and **L1Pt** can be promising candidates for treating liver, colon, breast, prostate, cervical, and brain cancers.

## 2. Results and Discussion

### 2.1. Synthesis and Characterization

**Scheme 1:**
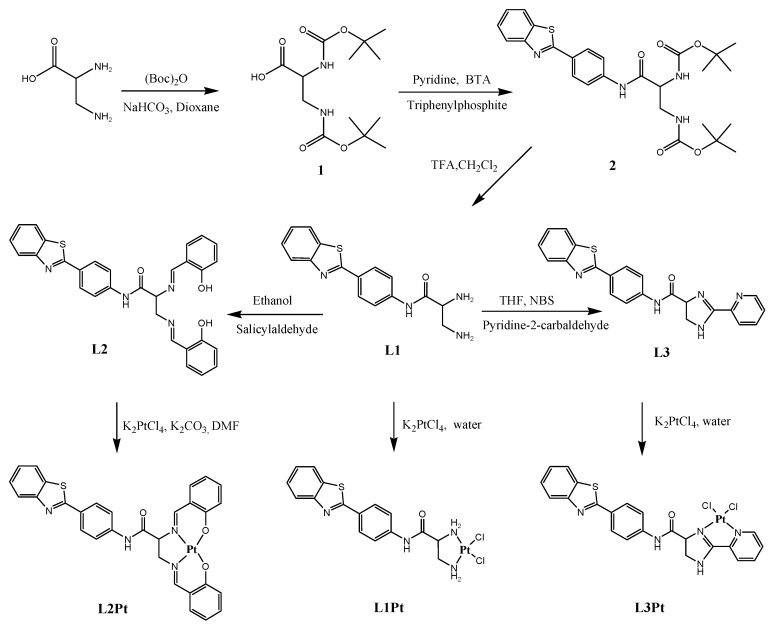
Synthesis of the benzothiazole aniline (BTA) derivatives and their platinum (II) complexes.

The syntheses of the BTA derivatives **L1**, **L2**, and **L3** and their Pt (II) complexes **L1Pt**, **L2Pt**, and **L3Pt** are depicted in Scheme 1. Compound **L1** was derived based on our previously published procedure [39], which was then condensed with two salicylaldehyde equivalents to give the **L2** ligand (white solid). The reaction of **L1** with 2-pyridinecarboxaldehyde at room temperature in the presence of N-bromosuccinamide resulted in **L3** (as a yellow solid). Platinum complexes **L1Pt**, **L2Pt**, and **L3Pt** were prepared by reacting the respective ligands **L1**, **L2**, and **L3** with an equivalent of K_2_PtCl_4_ under inert conditions. Potassium carbonate (K_2_CO_3_) was used to increase the reaction rate of complex **L2Pt**. The pure Pt (II) chelates were isolated in moderate yield after washing repeatedly with water, methanol and ether. The formation of the ligands and their Pt (II) complexes were confirmed using various spectroscopic methods such as ^1^H nuclear magnetic resonance (NMR), ^195^Pt-NMR, Fourier transform infrared spectroscopy (FTIR), high-resolution fast atom bombardment mass spectrometry (HR–FAB–MS), matrix-assisted laser desorption/ionization time-of-flight mass spectrometry (MALDI-TOF-MS), and elemental analysis (EA).

^1^H-NMR spectra of the newly synthesized ligands **L1**, **L2**, and **L3** and their Pt complexes **L1Pt**, **L2Pt**, and **L3Pt** were recorded in different solvents, such as MeOH-d_4_, CHCl_3_, DMSO-d_6_, and the details are presented in the experimental section. The ^1^H-NMR spectral data are consistent with the expected structure of the compounds (Supporting Information Figures S1, S4, S6, S9 and S12). In addition, complex **L1Pt** was characterized using ^195^Pt-NMR and the chemical shifts (δ = −2955 ppm, DMSO-d_6_), indicated no inter- or intramolecular contacts between the Pt (II) center and BTA moieties (Supporting Information Figure S7) [40]. The formation of **L2** was confirmed by the presence of a C=N stretching vibration at 1634 cm^−1^ and the coordination of the metal center was confirmed by the downshift of C=N at 1600 cm^−1^ due to the development of Ar–O–Pt–N=CH–chelating ring and loss of salicylic effect [41]. The FTIR spectrum of **L2** compared well to that of the corresponding **L2Pt** complex (Supporting Information Figures S3 and S10) Therefore, the ^1^H-NMR, ^195^Pt-NMR, and FTIR analysis data showed that the complexation of the ligand **L1** and **L2** with Pt was accomplished by coordination using amine and/or Schiff-bases nitrogen. In addition, high-resolution mass spectrometry in positive-ion mode was performed for all synthesized compounds. Parent peaks were found at (*m/z*) 578, (m/z) 714, and (*m/z*) 687 for complexes **L1Pt**, **L2Pt**, and **L3Pt** (Supporting Information Figures S8, S11 and S13). Ion peaks corresponding to the ligands have been observed at (*m/z*) 313, (*m/z*) 521, and (*m/z*) 400 for **L1**, **L2**, and **L3** (Supporting Information Figures S2 and S5) [39]. The purity of the newly synthesized compounds was confirmed by EA. **L1** was soluble in water and all other compounds when dissolved in DMSO.

### 2.2. Anticancer Effects and Cytotoxicity

**Figure 2:**
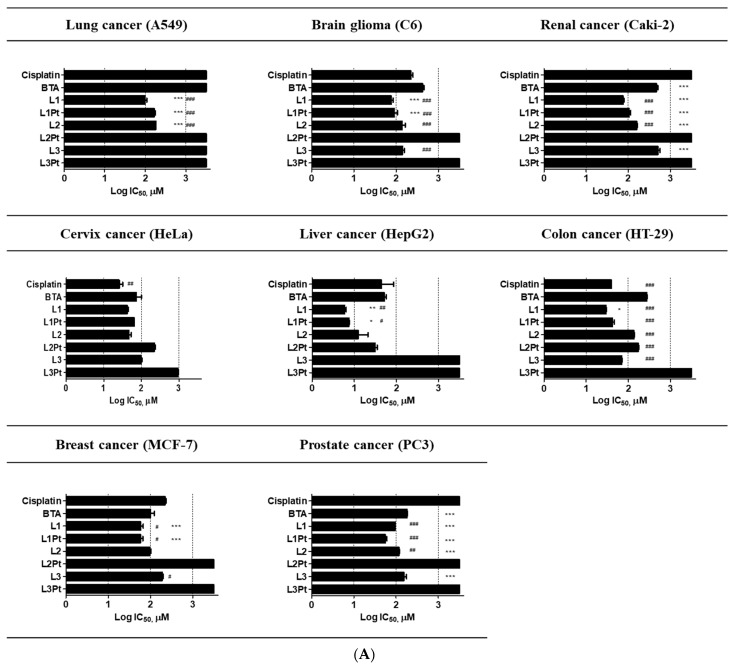

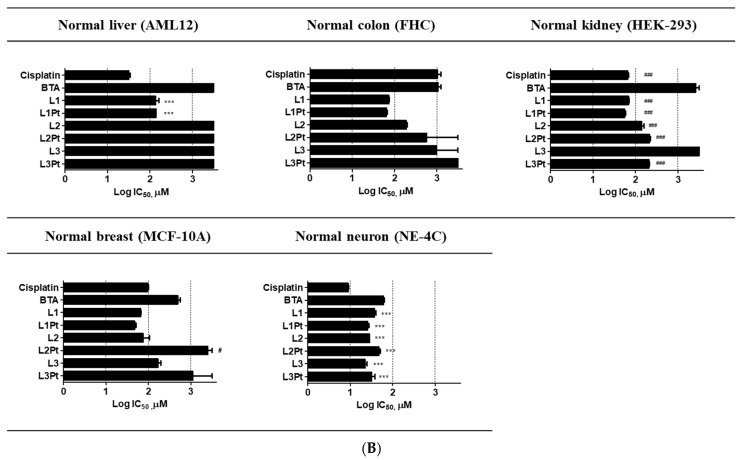
Toxicity profiles of anti-cancer agents. The graphs show the Log IC_50_ for each cytostatic agent in various cell lines. Log IC_50_ of at three independent experiments per cell line were averaged and summarized as a mean graph for better comparison of the different activities. (**A**) It shows the toxicity of synthesized compounds in various cancer cells. (**B**) It shows the toxicity of synthesized compounds in normal cell lines of various tissues. The red dotted line represents the cisplatin value, and the blue dotted line represents the BTA value. * *p* < 0.05, ** *p* < 0.01, *** *p* < 0.001, significant difference from the cisplatin. # *p* < 0.05, ## *p* < 0.01, ### *p* < 0.001, significant difference from the BTA.

We have tested the effects of different concentrations of ligands **L1**, **L2**, and **L3** and their platinum complexes **L1Pt**, **L2Pt**, and **L3Pt** on the viability of various cancerous and normal cell lines (Table 1 and Table 2, Figure 2). The half-inhibitory concentration (IC_50_) values were calculated based on these data. The data showed that each component inhibits the growth of different cell lines at varying degrees. **L1**, **L1Pt**, and **L2** exhibit similar cytotoxicity to that of cisplatin and BTA used as a control group in HeLa cells (Table 1 and Figure 2A). However, **L1**, **L1Pt**, and **L2** exhibited excellent toxicity to most of the cancer cells, except for HeLa cells. The **L1** and **L1Pt** exerted the best efficacy in liver and colon cancer cells. In addition, **L2Pt** revealed the anticancer effect only in liver cancer cells, and **L3** had the possibility of the anticancer effect in glioma and prostate cancer cells. Although **L3** induced a better anticancer effect than BTA in colon cancer cells, it did not reach the effect of cisplatin. **L3Pt** did not show anticancer effects in the tested cell lines, and the reason needs clarification. The structure–activity reveals that the conjugation of the 1,2-ethylenediamine with BTA (**L1** and **L1Pt**) exhibited excellent inhibitory activity against numerous cancer cells. Moreover, the exchange of the ethylene bridge with electron-donating hydroxyl group-containing phenol rings (i.e., compounds **L2** and **L2Pt**) displayed better anticancer activity than those of pyridine-containing derivatives **L3** and **L2Pt**. From toxicity comparison in normal cells (Table 2 and Figure 2B), we confirmed that the toxicity of **L1**, **L1Pt**, and **L2** was improved more than the cisplatin in the liver and brain cells. Although **L2Pt**, **L3**, and **L3Pt** did not exhibit toxicity in most of the normal cell lines, they did not display any anticancer effects, therefore, these compounds are not considered for further study.

### 2.3. Selective Antitumor Activity

A non-malignant mouse liver cell line (AML-12), human colon epithelial cells (FHC), human embryonic kidney cells (HEK-293), human breast epithelial cells (MCF-10A), and mouse brain neural stem-cell line (NE-4C) were used as models of the healthy cells to estimate the selectivity of these compounds in cancer cells regarding normal cells (Table 2, Figure 2B). To correctly term cytotoxicity as “selective” actually, there should be a great difference between tumor and non-tumor cells and it is challenging to compare cytotoxicity from different works. Interestingly, no cytotoxicity was observed for **L1**, **L2**, **L1Pt**, and **L2Pt** in normal mouse liver hepatocyte cells (AML12) up to 137 μM (Table 2). Moreover, compounds **L1, L2, L1Pt,** and **L2Pt** displayed an IC_50_ in liver cancer cells (HepG2) that are 23, 35, 18.5, and 15.8 folds higher in comparison with its IC_50_ value in normal liver cells (AML12) respectively [42]. In contrast, clinically used cisplatin exhibited an IC_50_ value in normal liver cells (AML12) 32 μM. In the case of liver cancer cells, **L1**, **L2**, **L1Pt**, and **L2Pt** showed strong toxicity, while revealed deteriorated toxicity in normal liver cells, demonstrate there is specificity for the liver cancer cells. In addition to liver cancer, **L1**, **L2**, **L3**, and **L1Pt** showed a more significant anticancer effect than cisplatin or BTA in brain glioma (Table 1). In the toxicity in normal cells, they have significantly improved toxicity than cisplatin in NE-4C cells (Table 2). We observed that compounds **L1**, **L2**, **L1Pt**, and **L2Pt** exhibited anticancer activity against colon cancer cells. In addition, compound **L3** displayed preferential anticancer activity against colon and breast cancer cells. This characteristic anticancer outcome may be attributed to the presence of the BTA moiety, which is well known for its tumor selectivity [23,24]. The superior selective toxicity toward cancer cells over noncancer cells proposes a strong potential of these compounds toward their antitumor application in liver cancer.

### 2.4. Stability of the Compounds in an Aqueous Solution

**Figure 3:**
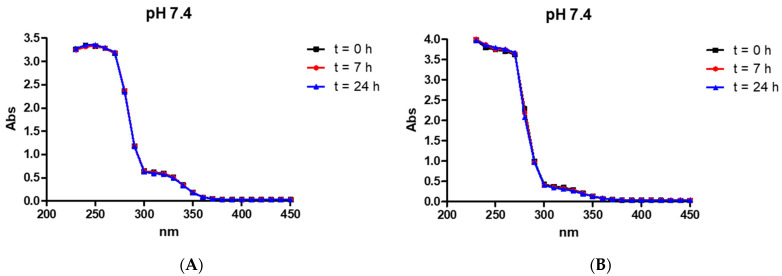
Time-dependent UV-Vis absorption spectra of compound **L1** (**A**) and **L1Pt** (**B**) in PBS solution recorded after t = 0 h (black line), t = 7 h (red line), and t = 24 h (blue line).

An indispensable feature of any promising anticancer agent is its thermodynamic stability in aqueous media. All prospective agents should be able to reach their target under the conditions encountered in living organisms. Therefore, the stability of the compounds was measured at 1 mM in phosphate-buffered saline (PBS) solution using UV–Vis spectroscopy. Due to poor solubility in aqueous media, compound **L1Pt** was dissolved in 2% DMSO in PBS solution. The stability of the metal complexes is often dependent on the pH value and the pH 4.5–5.0 is crucial since endosomal uptake of the complexes by lysosomes can occur [43]. Therefore, the UV–Vis spectrum was recorded in the range of 200–450 nm at t = 0, 7, and 24 h in different pH-values [(e.g., strong acidic (pH = 2), weakly acidic (pH = 5), neutral (pH = 7.4), alkaline (pH = 12)]. Only the most promising compounds were studied, and Figure 3 represents the time-dependent UV–Vis spectrum of the compound **L1** and **L1Pt** in physiological pH (7.4). The compounds expressed distinct peaks in the 200–450 nm region and did not display any significant changes throughout 24 h. Lack of substantial interactions in the absorptions peak and spectral characteristics for testing compounds over time propose that no structural alterations occurred in the aqueous solution [44,45]. Also, there were no obvious changes in the absorptions peak in other pH-values (such as acidic, weakly acidic, and alkaline) for complexes **L1** and **L1Pt** over time are shown in the Supporting Information (Figure S14).

### 2.5. Protein-Ligand Docking Simulation

The anticancer mechanism of platinum drugs involves their intercalation with DNA base pairs [46,47]. Thus, molecular docking studies were conducted to predict the binding poses of ligands in the active site of the DNA. Table 3 shows the docking binding energies of **L1**, **L1Pt**, and BTA with various DNA structures. The top-ranked binding energies (kcal/mol) in the Auto Dock output file were considered a response in every single run. The best docking result was considered with the lowest binding energy of the conformation. The predicted binding energies of compound **L1** and **L1Pt** were −6.697 and −7.150 kcal/mol, respectively, for binding to the 1BNA, whereas it was −6.658 kcal/mol for the parent compound BTA. Therefore, this negative binding energy suggests that those compounds rationally bind to DNA as their anticancer target. In addition, compounds **L1** and **L1Pt** with high cytotoxic activity also showed elevated binding energies, −5.839 and −5.695 kcal/mol, in binding to 3CO3, respectively. Compounds **L1** and **L1Pt** showed lower docking binding energy to DNA in binding to 1LU5 compared with parent BTA.

**Figure 4:**
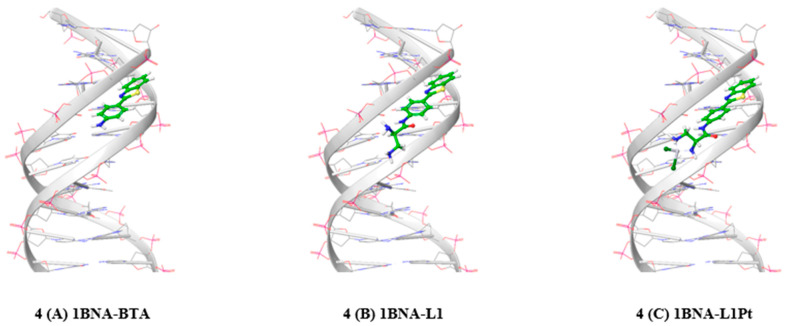
(**A**) Predicted binding pose of BTA on DNA (PDB ID: 1BNA). (**B**) Molecular docking simulation studies of the interaction between **L1** and DNA (PDB ID: 1BNA). (**C**) Molecular docking simulation studies of the interaction between **L1Pt** and DNA (PDB ID: 1BNA).

DNA intercalation and major or minor groove binding with DNA are the most frequently observed modes of interaction for small molecule drugs [48]. The molecular modeling results suggest that compounds **L1** and **L1Pt** interacted with the minor groove of the DNA (PDB ID: IBNA) (Figure 4). The BTA fragment of these compounds fits into the minor groove of the DNA and interacts through its sulfur group by forming hydrogen bonds with the base pairs. In addition, the –NH group showed a hydrogen bond interaction with the DNA. The docked poses of the compound **L1** and **L1Pt** (Figure 4) revealed that it binds to the minor groove of the DNA (PDB ID: 1BNA) using −6.697 and −7.150 kcal/mol binding energy. Cisplatin requires hydration to form diaqua species, which are considered active agents. However, in this study, **L1Pt** remains stable over 24 h and probably does not palatinate the DNA (Figure 3), nevertheless it acts by intercalation. In contrast, ligand **L1** showed groove binding intercalation with DNA and this can be one of the possible reasons for the higher cytotoxic effects than their corresponding Pt (II) complexes. The interactions of compound **L1** and **L1Pt** with the 3CO3 and 1LU5 structures of the DNA are shown in the Supporting Information [Figures S15 and S16]. However, we must mention that the docking study alone is not sufficient to probe mechanism of action of compounds. To probe it, at least in vitro binding assays such as ITC and SPR should be carried out on DNA- model systems, as well as on protein models. Therefore, further study using in vitro binding assays is warranted to probe mechanism of action of compounds.

## 3. Materials and Methods

### 3.1. Reagents and Instruments

Solvents were dried using standard methods. 2,3-Diaminopropionic acid, di-*tert*-butyl dicarbonate (Boc_2_O), and triphenylphosphite [P(OC_6_H_5_)_3_] were purchased from Tokyo Chemical Industry (Tokyo, Japan). Sodium bicarbonate (NaHCO_3_) and potassium carbonate (K_2_CO_3_) were purchased from Daejung Chem. (Siheung-si, Korea). Magnesium sulfate anhydrous (MgSO_4_) and sodium sulfate anhydrous (Na_2_SO_4_) were obtained from Duksan Scientific Corp. (Ansan-si, Korea). Salicylaldehyde was acquired from Junsei Chemical Co. Ltd. (Tokyo, Japan). 2-Pyridinecarboxaldehyde, 2-(4-aminophenyl)-benzothiazole, potassium tetra chloroplatinate (II) (K_2_PtCl_4_), and other commercial-grade reagents were purchased from Sigma-Aldrich (St. Louis, MO, USA), and used as received, unless otherwise stated. Deionized water (DI water) was used for the experiments. Progress of chemical reactions was observed using TLC (silica gel plates 60 F254) and visualized using a UV–Vis spectrometer. ^1^H-NMR experiments were performed using an Advance 500 spectrometer (Bruker, Billerica, MA, USA) at the instrumental analysis center of Kyungpook National University (KNU, Daegu, Korea). Chemical shifts (δ) are reported in ppm and coupling constants (*J*) in Hz. FTIR spectra were recorded using KBr pellets on a model 883 double beam infrared spectrophotometer (PerkinElmer, Waltham, MA, USA) in 200–4000 cm^−1^. Microanalysis was performed using a CHNS elemental analyzer (Thermo Fisher Scientific, Waltham, MA, USA) at the KNU instrumental analysis center. HR–FAB–MS spectra were recorded using a model JMS-700 spectrophotometer (JEOL, Tokyo, Japan) at the Korea Basic Science Institute. Complexation reactions were conducted under an inert atmosphere using standard Schlenk techniques. The purity of the synthesized compounds was confirmed by EA and the tested compounds had at least 95% purity.

### 3.2. Synthesis and Characterization

The syntheses of the benzothiazole aniline derivatives and their corresponding Pt (II) complexes are as follows:

#### 3.2.1. 2,3-Diamino-*N*-(4-benzothiazol-2-yl-phenyl)-propionamide (**L1**)

Compound **L1** was synthesized according to the previously reported method [39].

#### 3.2.2. *N*-(4-Benzothiazol-2-yl-phenyl)-2,3-bis-[(2-hydroxy-benzylidene)amino] propionamide (**L2**)

Salicylaldehyde (0.42 g, 3.5 mmol) was added dropwise to compound **L1** (0.54 g, 1.7 mmol) dissolved in ethanol (20 mL). The resulting mixture was refluxed around 3 h until the starting materials disappeared. Solids that appeared were filtered and rinsed with a small portion of cold ethanol, and pure product was harvested as a white solid after resuspending in hexane. Yield: 0.42 g (48%). ^1^H-NMR (500 MHz, CDCl_3_): δ = 12.8 (*s*, 2H, Ar-OH), 12.01 (*s*, 1H, NH), 8.45 (*s*, 1H, CH), 8.36 (*s*, 1H, CH), 8.07–8.01 (*m*, 3H, BTA), 7.90–7.86 (*d*, 1H, BTA), 7.71–7.66 (*d*, 2H, BTA), 7.49–7.44 (*t*, 1H, BTA), 7.41–7.38 (*t*, 1H, BTA), 7.36–7.25 (*m*, 3H, Ar-CH), 7.22–7.18 (*d*, 1H, Ar-CH), 7.04–6.81 (*m*, 4H, Ar-CH), 4.42–4.36 (*m*, 2H, CH_2_), 4.09–3.03 (*m*, 1H, CH). FTIR: v (cm^−1^) = 3299 w, 1665 s, 1634 (C=N) s, 1524 s, 1407 m, 1275 m, 967 m, and 749 m. HR–FAB–MS (*m/z*) for C_30_H_24_N_4_O_3_S: calcd, 521.1647 [M + H]^+^; found, 521.1649 [M + H]^+^. Anal. calcd for (C_30_H_24_N_4_O_3_S·½ H_2_O): C, 68.06; H, 4.76; N, 10.58; S, 6.06; found: C, 68.08; H, 4.59; N, 10.62; S, 6.24.

#### 3.2.3. 2-Pyridin-2-yl-4,5-dihydro-1*H*-imidazole-4-carboxylic acid (4-benzothiazol-2-yl-phenyl)amide (**L3**)

2-Pyridinecarboxaldehyde (0.54 mL, 5.06 mmol) was added to a solution of compound **L1** (1.58 g, 5.06 mmol) in dry tetrahydrofuran (40 mL) using an ice bath. *N*-Bromosuccinimide (0.52 g, 2.94 mmol) in THF was added dropwise and stirred overnight at room temperature (RT). The resulting mixture was extracted using saturated NaHCO_3_ solution and washed with CH_2_Cl_2_. The collected organic layer was rinsed using saturated NaCl and dried over Na_2_SO_4_. After precipitation pure product was collected as a pale-yellow solid. Yield: 0.7 g (36%). ^1^H-NMR (500 MHz, CDCl_3_): δ = 9.82 (*s,* 1H, NH), 9.46 (*s*, 1H, NH), 8.54 (*s*, 1H, CH-Py), 8.33 (*dd*, 1H, CH-Py), 8.23 (*s*, 1H, C–Py), 7.81 (*m*, 2H, BTA), 7.62 (*s*, 1H, BTA), 7.54 (*t*, 3H, BTA), 7.29–7.20 (*dd*, 2H, BTA), 7.06 (*t*, 1H, CH-Py), 4.86 (*s*, 1H, CH), 4.16–3.74 (*d*, 2H, CH_2_). Anal. Calcd for (C_22_H_17_N_5_OS.1.5 H_2_O): C, 61.98; H, 4.71; N, 16.42; S, 7.52; found: C, 62.32; H, 3.98; N, 15.64; S, 7.54. HR–FAB–MS (*m/z*) for C_23_H_17_N_5_OS: Calcd, 400.1232 [M + H]^+^; found: 400.1233 [M + H]^+^.

#### 3.2.4. Synthesis of Complex **L1Pt**

A solution of K_2_PtCl_4_ (0.14 g, 0.35 mmol) in distilled water (10 mL) was prepared under an inert atmosphere and added to compound **L1** (0.11 g, 0.35 mmol). The resultant mixture was stirred overnight in the dark at RT. The precipitate obtained was filtered and rinsed using water, methanol, and ethyl ether. Vacuum drying produced a pale-yellow solid. A characteristic peak was observed at −2754 ppm corresponding to the Pt (II) species. Yield: 0.10 g (53%). ^1^H-NMR (500 MHz, DMSO-d_6_): δ = 11.03 (*w*, 1H, NH), 8.77–8.51 (*w*, 2H, NH), 8.17–7.99 (*m*, 4H, BTA), 7.92–7.80 (*d*, 2H, BTA), 7.55 (*t*, 1H, BTA), 7.46 (*t*, 1H, BTA), 6.62–6.20 (*dd*, 2H, NH_2_), 3.83 (*dd*, 1H, CH), 3.02–2.72 (*dd*, 2H, CH_2_). ^195^Pt-NMR (DMSO-d_6_): δ = −2754. Anal. calcd for (C_16_H_16_Cl_2_N_4_OPtS∙2.5H_2_O): C, 30.84; H, 3.39; N, 8.99; S, 5.15; found: C, 30.62; H, 2.81; N, 8.74; S, 5.20. HR–FAB–MS (*m/z*) for C_16_H_16_Cl_2_N_4_OPtS: calcd, 578.0148 [M + H]^+^; found, 578.0144 [M + H]^+^.

#### 3.2.5. Synthesis of Complex **L2Pt**

Potassium carbonate (0.19 g, 1.35 mmol) was added to a solution of ligand **L2** (0.35 g, 0.67 mmol) in DMF (10 mL). Next a solution of potassium tetrachloroplatinate (0.28 g, 0.67 mmol) in water (10 mL) was prepared under a stream of nitrogen and added to the mixture, which was then stirred overnight at a reaction temperature lower than 60 °C. The reaction mixture was left to cool at RT and the solid was filtered. Pure compound was gathered as a pale-yellow solid following repeated flushing with water, methanol, and ethyl ether. Yield: 0.24 g (49%). ^1^H-NMR (500 MHz, DMSO-d_6_): δ = 10.75 (*s*, 1H, NH), 8.76 (*s*, 1H, Ar-CH), 8.60 (*s*, 1H, Ar-CH), 8.17–7.78 (*m*, 6H, BTA), 7.69–7.37 (*d*, 6H, Ar-CH), 6.95 (*t*, 1H, BTA), 6.65 (*t*, 1H, BTA), 4.75 (*m*, 1H, CH), 4.32 (*d*, 1H, CH_2_), 4.15 (*d*, 1H, CH_2_), 2.09 (*s*, 2H, NH_2_). FTIR: v (cm^−1^) = 3054 w, 1693 s, 1600 s (C=N), 1532 s, 1437 m, 1302 m. Anal. calcd for (C_30_H_22_N_4_O_3_PtS·2H_2_O): C, 48.06; H, 3.50; N, 7.47; S, 4.28; found: C, 48.10; H, 3.59; N, 7.29; S, 6.87. HR–FAB–MS (*m/z*) for C_30_H_22_N_4_O_3_PtS: calcd, 714.1139 [M + H]^+^; found, 714.1142 [M + H]^+^.

#### 3.2.6. Synthesis of Complex **L3Pt**

A solution of K_2_PtCl_4_ (0.45 g, 1.10 mmol) in distilled water (15 mL) was prepared under a stream of nitrogen and added to a solution of ligand **L3** (0.4 g, 1.10 mmol) in ethanol (15 mL). The resultant mixture was stirred in the dark under a nitrogen atmosphere at 50 °C, overnight. Solid that appeared was filtered and the pale-yellow product harvested after repeated washing using water, ethanol, and Et_2_O. Yield: 0.27 g (39%). ^1^H- NMR (500 MHz, DMSO-d_6_): δ = 8.17–7.98 (*m*, 6H, BTA), 7.86 (*t*, 1H, BTA), 7.82 (*t*, 1H, BTA), 7.57–7.42 (*m*, 4H, Py), 5.04 (*t*, 1H, CH), 4.56–3.98 (*m*, 2H, CH_2_). Anal. calcd for (C_22_H_17_Cl_2_N_5_OPtS·2H_2_O): C, 37.67; H, 3.02; N, 9.98; S, 4.57; found: C, 37.81; H, 2.71; N, 8.97; S, 3.57. HR-MS (*m/z*) for C_22_H_17_Cl_2_N_5_OPtS: calcd, 687.0076 [M + Na]^+^; found, 687.2321 [M + Na]^+^.

### 3.3. Stability of the Compounds in Aqueous Solution

The stability of the most active compounds, **L1** and **L1Pt**, was studied. The compounds, **L1** and **L1Pt**, at a concentration of 1 mM in phosphate-buffered saline (PBS) solution at different pH-values [(e.g., strong acidic (pH = 2), weakly acidic (pH = 5), neutral (pH = 7.4), alkaline (pH = 12)] were evaluated using UV–Vis spectroscopy. In the case of **L1Pt**, 2% DMSO in PBS was used as solvent due to the poor water solubility. The spectra were recorded in the range of 200–450 nm at t = 0, 7, and 24 h, and compared to each other.

### 3.4. Cell Culture

Human colorectal adenocarcinoma cells (HT-29, ATCC^®^ HTB-38), adenocarcinomic human alveolar basal epithelial cells (A549, ATCC^®^ CCL-185), and prostate cancer (PC-3, ATCC^®^ CRL-1435) were cultured in the growth medium, containing Roswell Park Memorial Institute Medium (RPMI1640) WelGENE, Daegu, Korea supplemented with 10% (*v/v*) fetal bovine serum (FBS, Gibco, Grand Island, NY, USA) and 1% antibiotics-antimycotics (Gibco). Human breast adenocarcinoma cells (MCF-7, ATCC^®^ HTB-22™), hepatocellular carcinoma (HepG2, ATCC^®^ HB-8065™), *Rattus* brain glioma (C6, ATCC ^®^ CRL-2303™), and human renal carcinoma (Caki-2, ATCC^®^ HTB-47™) cells were cultured in Dulbecco’s modified Eagle’s medium (DMEM, WelGENE, Daegu, Korea) supplemented with 10% (*v*/*v*) FBS (Gibco) and 1% antibiotics-antimycotics (Gibco). Mouse brain neural (NE-4C, ATCC^®^ CRL-2925™) and human cervix adenocarcinoma cells (HeLa, ATCC^®^ CCL-2™) were cultured in Eagle’s minimum essential medium (EMEM, ATCC^®^ 30-2003™) supplemented with 10% FBS (Gibco) and 1% antibiotics-antimycotics (Gibco). Mouse liver normal cells (AML12, ATCC^®^ CRL-2254™) were cultured in DMEM/F-12 (WelGENE) supplemented with 10% FBS (Gibco), 1 × ITS (10 μg/mL insulin, 5.5 μg/mL transferrin, 6.7 ng/mL selenium, Gibco), 40 ng minimum essential medium Eagle (WelGENE) supplemented with 10% FBS (Gibco, USA) and 1% antibiotics-antimycotics (Gibco). Human breast epithelial cells (MCF 10A, ATCC^®^ CRL-10317™) were cultured in DMEM/F-12 (WelGENE) supplemented with 5% horse serum (Gibco), 20 ng/mL epidermal growth factor (EGF, Peprotech Inc., Rocky Hill, NJ, USA), 500 μg/mL hydrocortisone (Sigma-Aldrich, St. Louis, MO, USA), 100 ng/mL colera toxin (Sigma-Aldrich), 10 μg/mL insulin (Sigma-Aldrich) and 1% antibiotics- (Gibco). Human colon normal epithelial cells (FHC, ATCC^®^ CRL-18317™) were cultured in DMEM/F-12 (WelGENE) supplemented with 10% FBS (Gibco), 10 ng/mL cholera toxin (Sigma-Aldrich), 20 ng/mL EGF (Peprotech Inc.), 100 μg/mL hydrocortisone (Sigma-Aldrich), 1 × ITS (10 μg/mL insulin, 5.5 μg/mL transferrin, 6.7 ng/mL selenium, Gibco) and 1% antibiotics-antimycotics (with 100 units penicillin, 100 μg streptomycin and 250 ng amphotericin B per mL, Gibco). Cells were incubated in a humidified 5% CO_2_ atmosphere at 37 °C.

### 3.5. Cell Viability Assay

To evaluate cytotoxicity, cells were seeded in a 96-well plate (FHC, 0.5 × 10^4^ cells/well; Caki-2 and MCF 10A, 1 × 10^4^ cells/well; AML12, 1.2 × 10^4^ cells/well; HeLa, HEK-293, A549, and PC-3, 1.5 × 10^4^ cells/well; HT-29, MCF-7, C6, HepG2, and NE-4C, 2 × 10^4^ cells/well). After attaching and stabilizing cells for 24 h, the medium was switched to various concentrations (0, 2.5, 5, 10, 20, 30, 40, 50, 100, and 200 μM) of cisplatin (Sigma-Aldrich), BTA (Sigma-Aldrich), **L1**, **L1Pt**, **L2**, **L2Pt**, **L3**, and **L3Pt**, and cells were incubated for 22 h. In addition, cell counting kit-8 (CCK-8, Dojindo Laboratories, Kumamoto, Japan) solution was added to each well, and the plate was incubated for 2 h. The absorbance was then measured at 450 nm using a microplate reader (SpectraMax i3, Molecular Devices, San Jose, CA, USA). The IC_50_ and logIC_50_ values were calculated in GraphPad Prism (GraphPad Prism Software Inc. Version 5.02, San Diego, CA, USA). All experiments were performed, independently, three times. The graph of logIC_50_ values represents the average value.

### 3.6. Molecular Docking

Binding poses and energies of **L1**, **L1Pt**, and BTA for DNA structures were predicted using a protein-ligand docking simulation application called Glide [49]. Glide searches for possible binding poses of given ligands on DNA structure surface and finds the best binding poses and energy using the empirical scoring function called GlideScore [49]. For docking simulation, the OPLS3 force field was used to describe the atomic forces of ligand and DNA molecules. Flexible ligand sampling of ligand was allowed and the standard precision mode of Glide was used.

### 3.7. Statistical Analysis

Data were evaluated using a one-way analysis of variance with Tukey’s multiple comparison tests. Analyses were performed using GraphPad Prism (GraphPad Prism Software Inc., version 5.02). Data are expressed as mean ± SD (standard deviation) or standard error of the mean values, and *p* < 0.05 was considered significant, statistically.

## 4. Conclusions

In this study, three novel benzothiazole aniline derivatives **L1**, **L2**, and **L3** and their corresponding Pt (II) complexes **L1Pt**, **L2Pt**, and **L3Pt** have been designed and synthesized. The targeted compounds were investigated for their in vitro cytotoxic activity using the CCK-8 assay against various cancer and normal cells lines. Compared to the parental BTA and clinically used cisplatin, compounds **L1**, and **L1Pt** demonstrated selective inhibitory activities against liver cancer cells. In addition, docking results indicate that compounds **L1** and **L1Pt** interact with the minor groove of the DNA, and remain stable in aqueous media. Therefore, these compounds may be considered as prospective alternatives to the present chemotherapy drugs.

## Figures and Tables

**Table 1:** Half inhibition concentrations (IC_50_) of synthesized compounds in cancer cells. Various cancer cells treated with different concentrations of compounds were assayed for cell viability by the CCK-8 method at 24 h and IC_50_ values were calculated using the GraphPad Prism 5 software. IC_50_ values for compound against each cell line are means of three independent experiments [(*n* = 3, mean ± SEM (standard error of mean)]. Cisplatin and BTA, which are the backbones of the compounds, were compared as positive controls.

Tissue Sources	Cell Lines	IC_50_ (μM) for							
		Cisplatin	BTA	L1	L1Pt	L2	L2Pt	L3	L3Pt
Human lung cancer	A549	>500	>500	100.16 ± 8.4	167.2 ± 11.2	182.4 ± 0.8	>500	>500	>500
Human brain glioma	C6	226.6 ± 34.3	424.7 ± 47.7	77.8 ± 9.7	93.3 ± 22.7	143.2 ± 32.7	>500	142.4 ± 15.3	>500
Human renal cancer	Caki-2	>500	462.8 ± 44.1	75.6 ± 3.4	105.7 ± 8.5	159.0 ± 4.3	>500	>500	>500
Human cervical cancer	HeLa	26.2 ± 5.6	76.4 ± 23.4	41.9 ± 3.4	64.7 ± 0.3	47.0 ± 6.0	221.0 ± 15.8	98.9 ± 3.5	>500
Human hepatic carcinoma	HepG2	54.2 ± 31.8	52.0 ± 5.7	5.9 ± 0.5	7.5 ± 0.1	14.2 ± 7.0	31.6 ± 3.6	>500	>500
Human colon cancer	HT-29	39.1 ± 0.6	275.3 ± 5.0	29.9 ± 0.3	42.6 ± 5.6	135.0 ± 4.4	172.6 ± 3.2	70.5 ± 0.2	>500
Human breast cancer	MCF-7	203.8 ± 27.3	103.9 ± 28.6	65.9 ± 12.3	59.0 ± 11.0	98.1 ± 7.9	>500	190.3 ± 16.5	>500
Human prostate cancer	PC-3	>500	181.7 ± 7.8	94.9 ± 0.8	55.7 ± 7.5	116.3 ± 5.0	>500	130.2 ± 34.7	>500

**Table 2:** Half inhibition concentration (IC_50_) of synthesized compounds in normal cells. Various normal cells treated with different concentrations of compounds were assayed for cell viability by the CCK-8 method at 24 h and IC_50_ values were calculated using the GraphPad Prism 5 software. IC_50_ values for compound against each cell line are means of three independent experiments (*n* = 3, mean ± SEM). Cisplatin and BTA, which are the backbones of the compounds, were compared as positive controls.

Tissue Sources	Cell Lines	IC_50_ (μM) for							
		Cisplatin	BTA	L1	L1Pt	L2	L2Pt	L3	L3Pt
Mouse liver hepatocyte	AML12	32.1 ± 4.4	>500	137.6 ± 24.1	139.0 ± 0.8	>500	>500	>500	>500
Human colon epithelial	FHC	>500	>500	72.7 ± 2.0	64.3 ± 2.4	189.2 ± 10.3	>500	>500	>500
Human embryonic kidney	HEK-293	65.5 ± 4.1	>500	68.1 ± 2.8	54.9 ± 2.8	138.9 ± 20.1	211.5 ± 14.9	>500	205.9 ± 7.4
Human breast epithelial	MCF-10A	95.9 ± 6.7	401.4 ± 136.3	65.1 ± 1.9	46.9 ± 5.1	79.3 ± 25.4	>500	>500	>500
Mouse brain neural stem cell	NE-4C	9.0 ± 0.3	60.9 ± 2.9	36.8 ± 3.7	25.7 ± 1.7	28.2 ± 0.1	47.0 ± 4.1	22.4 ± 2.4	32.4 ± 6.0

**Table 3:** Predicted binding energies of **L1**, **L1Pt**, and BTA on different DNA structures.

Docking Binding Energy (kcal/mol)			
Name	1BNA	3CO3	1LU5
L1	−6.697	−5.839	−4.636
L1Pt	−7.150	−5.695	−4.164
BTA	−6.658	−6.495	−5.037

## Data Availability

Data is contained within the article and supplementary files.

## Supplementary Materials

The following are available online at https://www.mdpi.com/article/10.3390/ph14080832/s1, **Content:** Figure S1: ^1^H-NMR spectrum of compound **L2**, Figure S2: High resolution-FAB- mass spectrum of compound **L2**, Figure S3: FTIR spectrum of compound **L2**, Figure S4: ^1^H-NMR spectrum of compound **L3**, Figure S5: High resolution-FAB- mass spectrum of compound **L3**, Figure S6: ^1^H-NMR spectrum of compound **L1Pt**, Figure S7: ^195^Pt-NMR spectrum of compound **L1Pt**, Figure S8: High resolution-FAB- mass spectrum of compound **L1Pt**, Figure S9: ^1^H-NMR spectrum of compound **L2Pt**, Figure S10: FTIR spectrum of compound **L2Pt**, Figure S11: High resolution-FAB- mass spectrum of compound **L2Pt**, Figure S12: ^1^H-NMR spectrum of compound **L3Pt**, Figure S13: MALDI-TOF mass spectrum of compound **L3Pt**, Figure S14. Time-dependent UV-Vis absorption spectra of **L1** and **L1Pt**. Figure S15: Molecular docking of BTA, **L1**, and **L1Pt** with DNA, Figure S16: Molecular docking of BTA, **L1**, and **L1Pt** with DNA.

## Abbreviations

BTA	Benzothiazole aniline
RT	Room temperature
CCK-8	Cell counting kit-8
DNA	Deoxyribonucleic acid
DI	Deionized
SAR	Structure-activity relationship
FTIR	Fourier-transform infrared spectroscopy
MALDI-TOF	Matrix-assisted laser desorption/ionization time-of-flight mass spectrometry
PBS	Phosphate-buffered solution
UV	Ultraviolet
DMSO	Dimethyl sulfoxide
NMR	Nuclear magnetic resonance
