# Supplementary material for: Synthesis, Characterization, and Anticancer Activity of Benzothiazole Aniline Derivatives and Their Platinum (II) Complexes as New Chemotherapy Agents
Source: Pharmaceuticals (Basel). 2021 Aug 23;14(8):832. doi: 10.3390/ph14080832 (PMC8399196; doi:10.3390/ph14080832)
Supplement: Supplementary file 1 [file pharmaceuticals-14-00832-s001.zip › pharmaceuticals-1340931-supplementary.pdf]

Supplementary Files

Synthesis, Characterization, and Anticancer Activity of Benzothiazole Aniline Derivatives and Their Platinum (II) Complexes as a New Chemotherapy Agents

Md. Kamrul Islam [1], Ah-Rum Baek [2], Bokyung Sung [2], Byeong-Woo Yang [2], Garam Choi [3], Hyun-Jin Park [3], Yeoun-Hee Kim [3], Minsup Kim [4], Seongmin Ha [5], Gang-Ho Lee [6], Hee-Kyung Kim [7,*] and Yongmin Chang [2,8,9,*]

1. Institute of Biomedical Engineering Research, Kyungpook National University, 680, Gukchaebosang-ro, Jung-gu, Daegu 41944, Korea
2. Department of Medical & Biological Engineering, Kyungpook National University, 80, Daehak-ro, Buk-gu, Daegu 41566, Korea
3. R&D Center, Mirae BioPharm. Co., 124, Sagimakgol-ro, Jungwon-gu, Gyeonggi-do, 13207, Korea
4. InCerebro Drug Discovery Institute, 01811, Seoul, Korea
5. Department of Medical Science, School of Medicine, Kyungpook National University, 680, Gukchaebosang-ro, Jung-gu, Daegu 41944, Korea
6. Department of Chemistry, Kyungpook National University, 80, Daehak-ro, Buk-gu, Daegu 41566, Korea
7. Laboratory Animal Center, the Daegu-Gyeongbuk Medical Innovation Foundation, 88 Dongnae-ro, Dong-gu, Daegu 41061, Korea
8. Department of Molecular Medicine, School of Medicine, Kyungpook National University, 680, Gukchaebosang-ro, Jung-gu, Daegu 41944, Korea
9. Department of Radiology, Kyungpook National University Hospital, 130 Dongdeok-ro, Jung-gu, Daegu 41944, Korea

* Correspondence: ychang@knu.ac.kr; Tel.: (+) 82-53-420-5471 (Y. C.); hkkim@dgmif.re.kr (H. K.K.)

Contents

1.	**Figure S1**. ^{1}H NMR spectrum of compound **L2**.	S3
2.	**Figure S2**. High resolution-FAB- mass spectrum of compound **L2**.	S3
3.	**Figure S3**. FTIR spectrum of compound **L2**.	S4
4.	**Figure S4**. ^{1}H NMR spectrum of compound **L3**.	S5
5.	**Figure S5**. High resolution-FAB- mass spectrum of compound **L3**.	S5
6.	**Figure S6**. ^{1}H NMR spectrum of compound **L1Pt**.	S6
7.	**Figure S7**. ^{195}Pt NMR spectrum of compound **L1Pt**.	S6
8.	**Figure S8**. High resolution-FAB- mass spectrum of compound **L1Pt**.	S7
9.	**Figure S9**. ^{1}H NMR spectrum of compound **L2Pt**.	S7
10.	**Figure S10**. FTIR spectrum of compound **L2Pt**.	S8
11.	**Figure S11**. High resolution-FAB- mass spectrum of compound **L2Pt**.	S8
12.	**Figure S12**. ^{1}H NMR spectrum of compound **L3Pt**.	S9
13.	**Figure S13**. Maldi-tof mass spectrum of compound **L3Pt**.	S9
14.	**Figure 14**. Time-dependent UV-Vis absorption spectra of **L1** and **L1Pt**.	S10
15.	**Figure 15**. Molecular docking of BTA, **L1**, and **L1Pt** with DNA.	S10
16.	**Figure 16**. Molecular docking of BTA, **L1**, and **L1Pt** with DNA.	S11

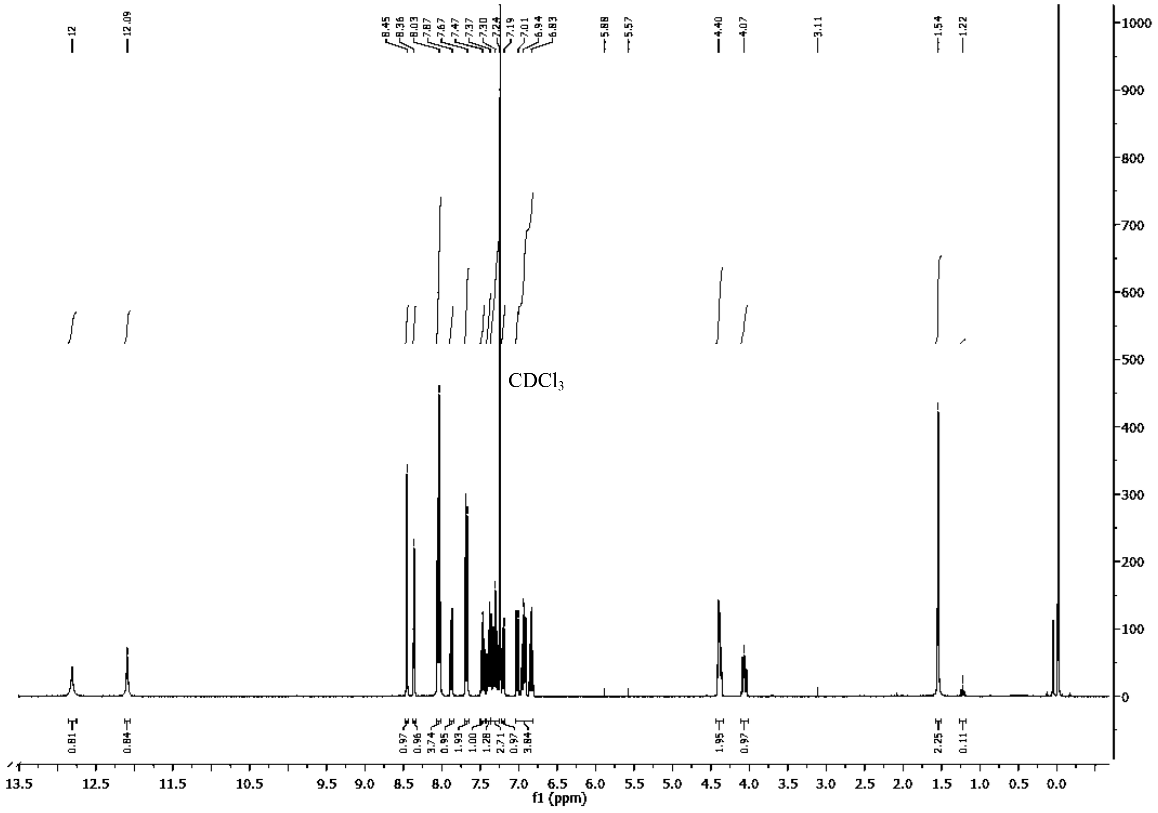

Figure S1. ^{1}H NMR spectrum of compound **L2**.

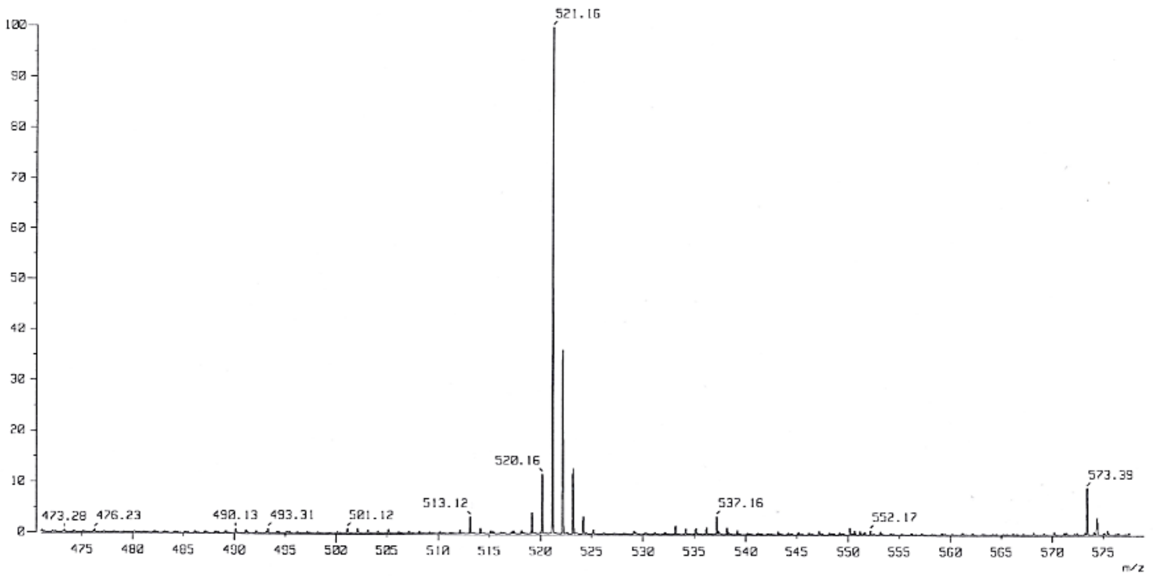

Figure S2. High resolution-FAB- mass spectrum of compound **L2**.

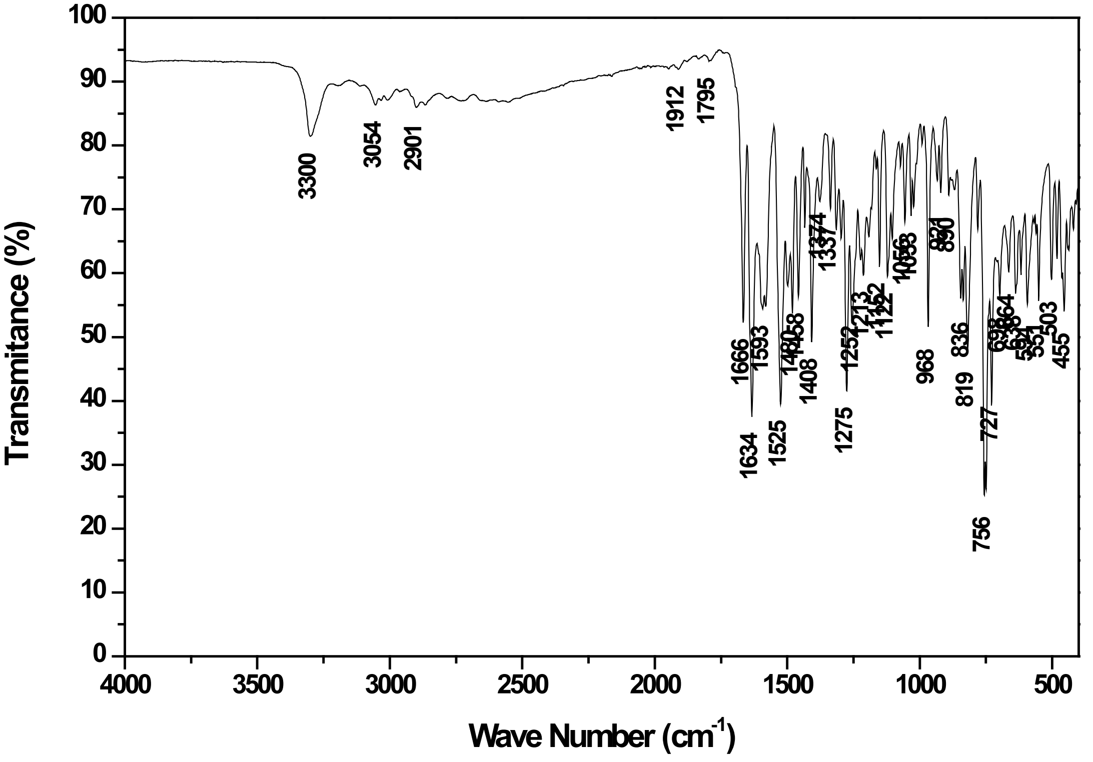

Figure S3. FTIR spectrum of compound **L2**.

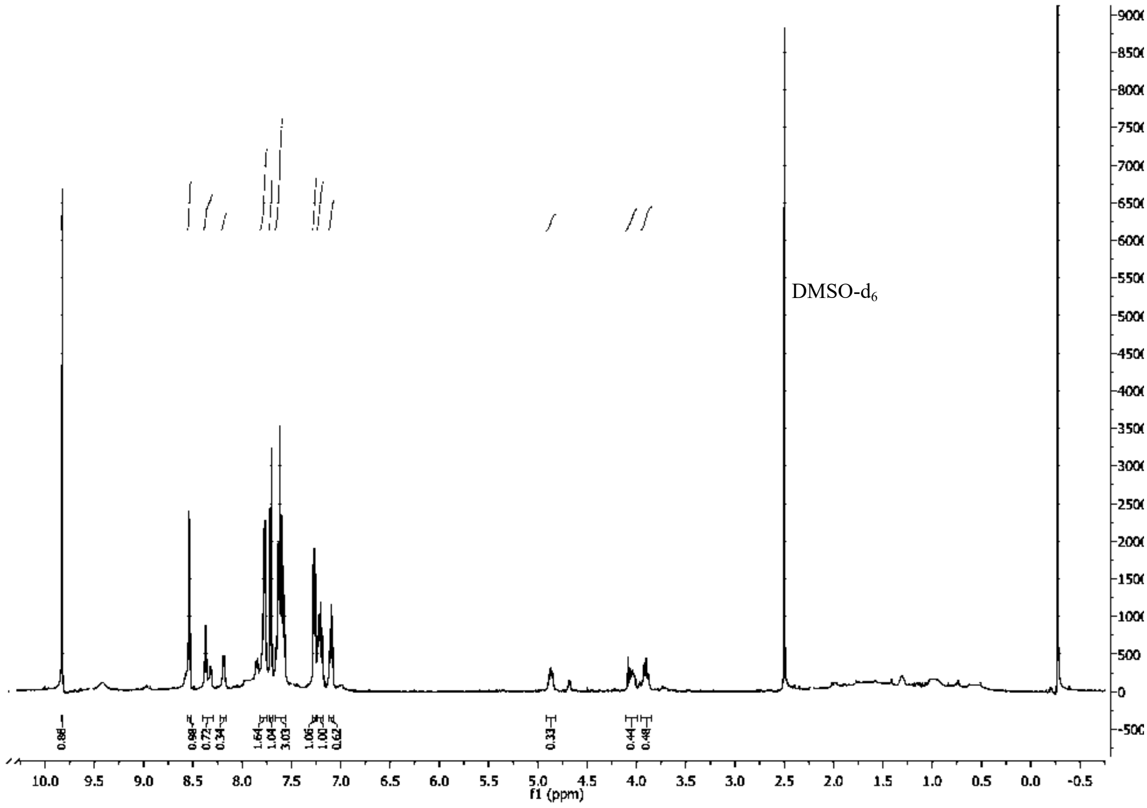

Figure S4. ^{1}H NMR spectrum of compound **L3**.

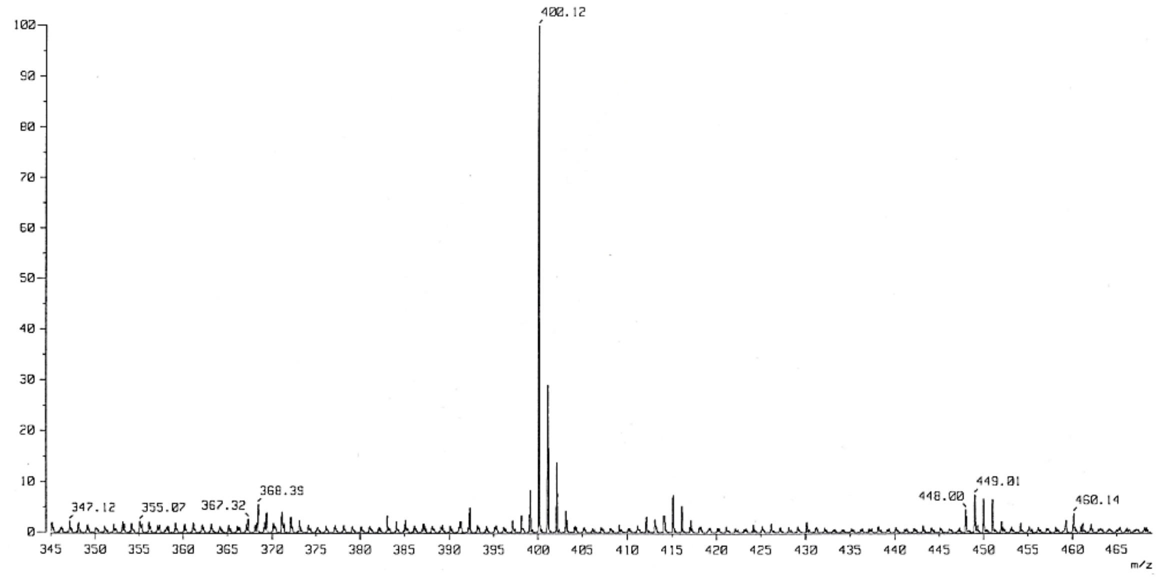

Figure S5. High resolution-FAB- mass spectrum of compound **L3**.

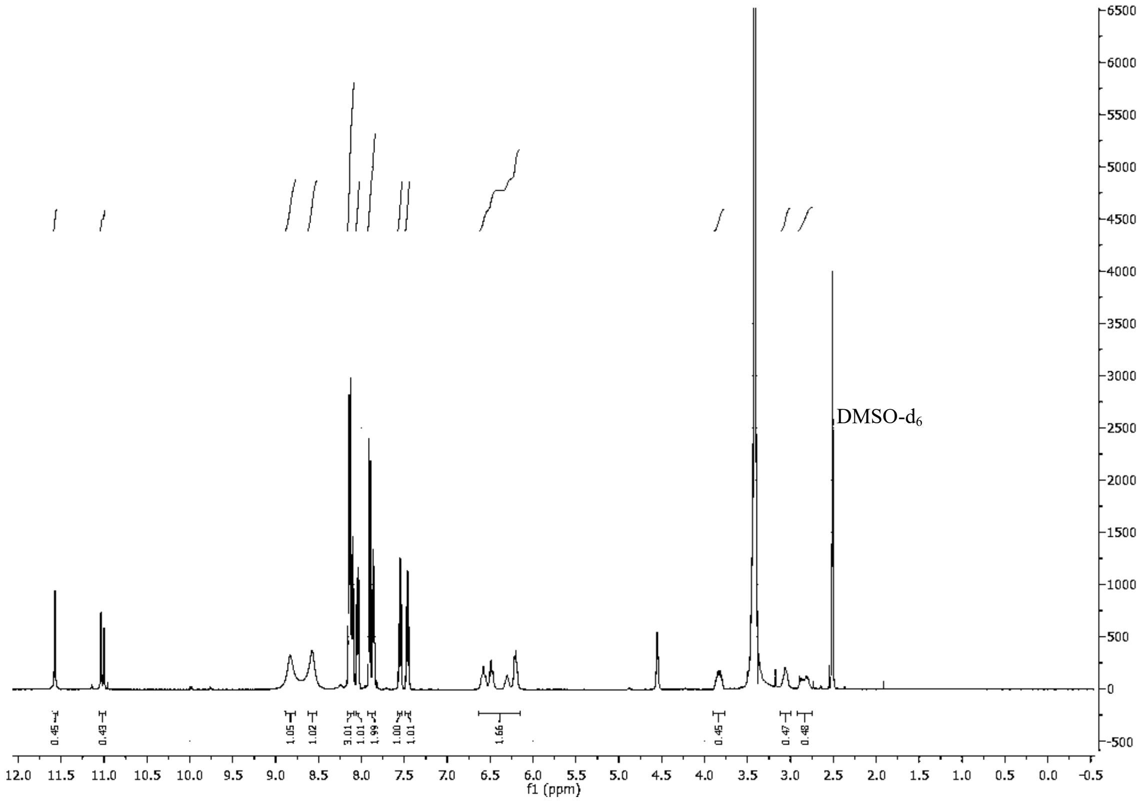

Figure S6. ^{1}H NMR spectrum of compound **L1Pt**.

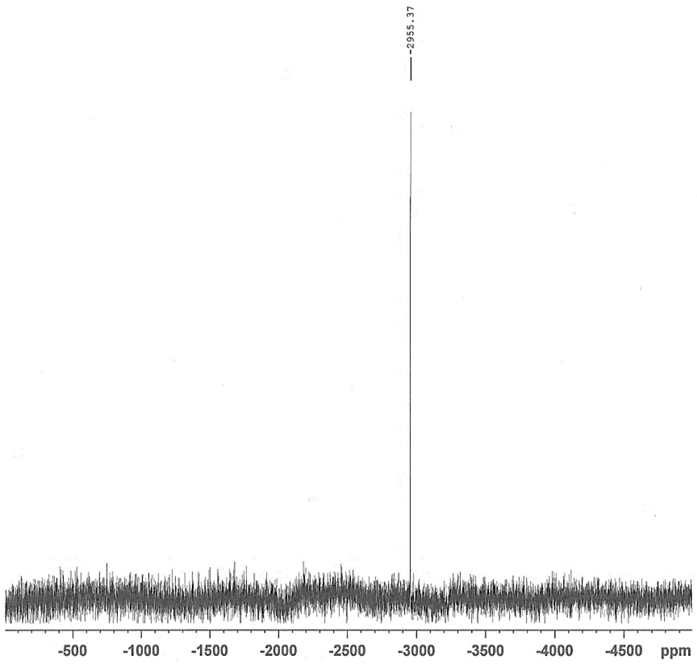

Figure S7. ^{195}Pt NMR chemical shift (ppm) of compound **L1Pt**.

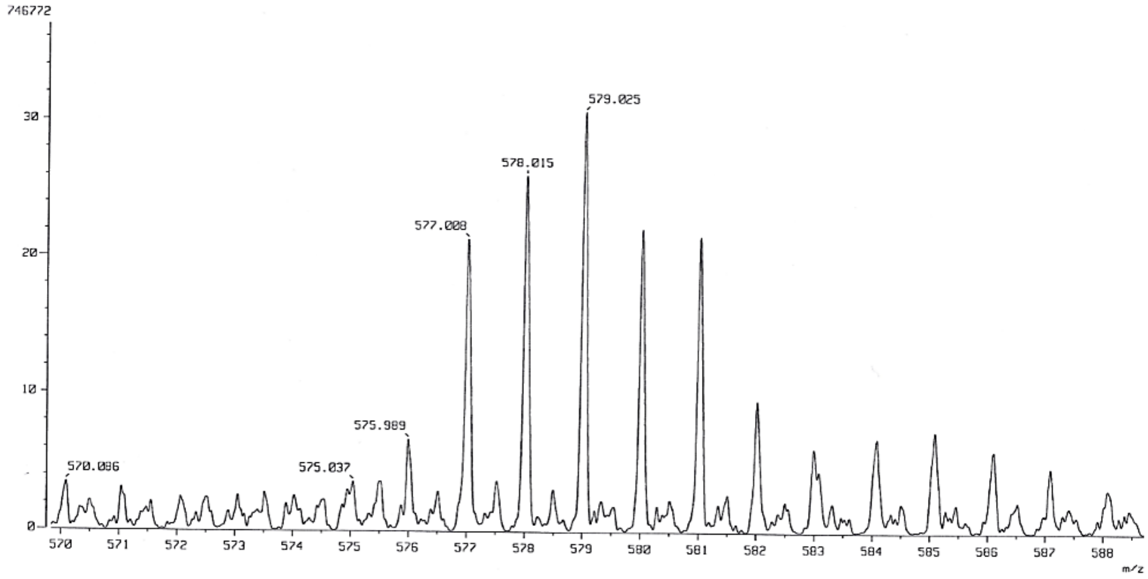

Figure S8. High resolution-FAB- mass spectrum of Compound **L1Pt**.

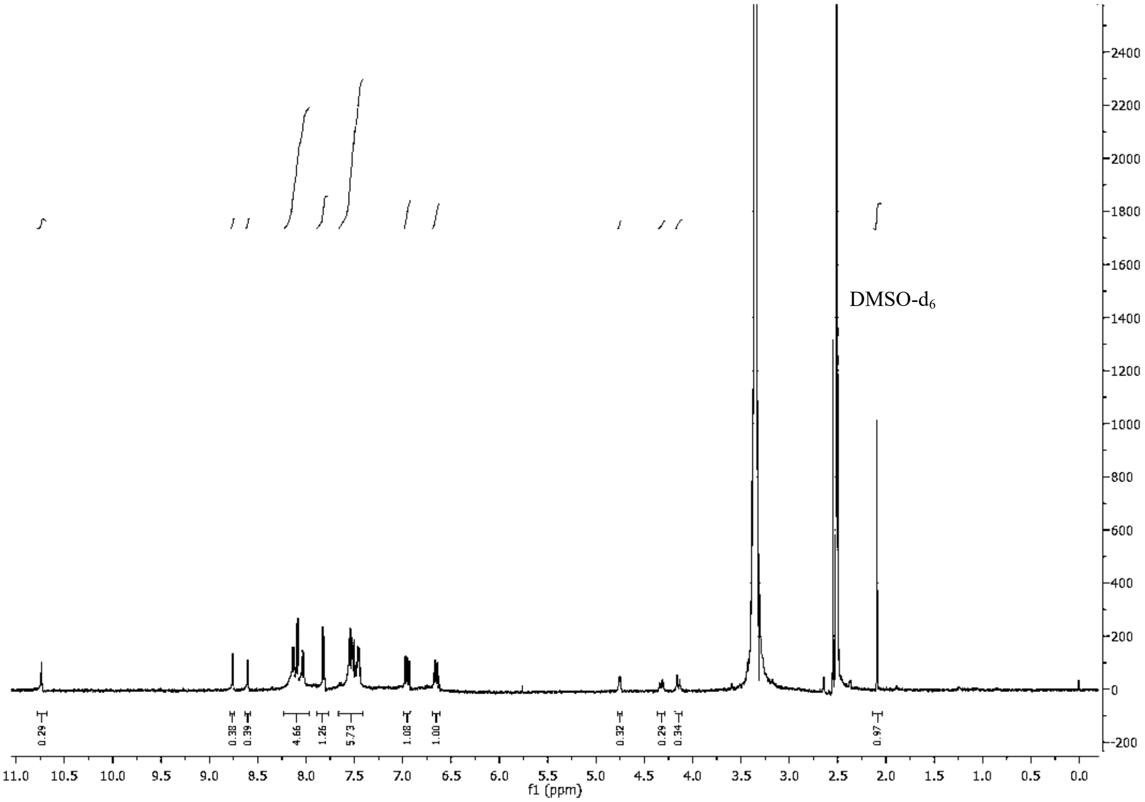

Figure S9. ^{1}H NMR spectrum of compound **L2Pt**.

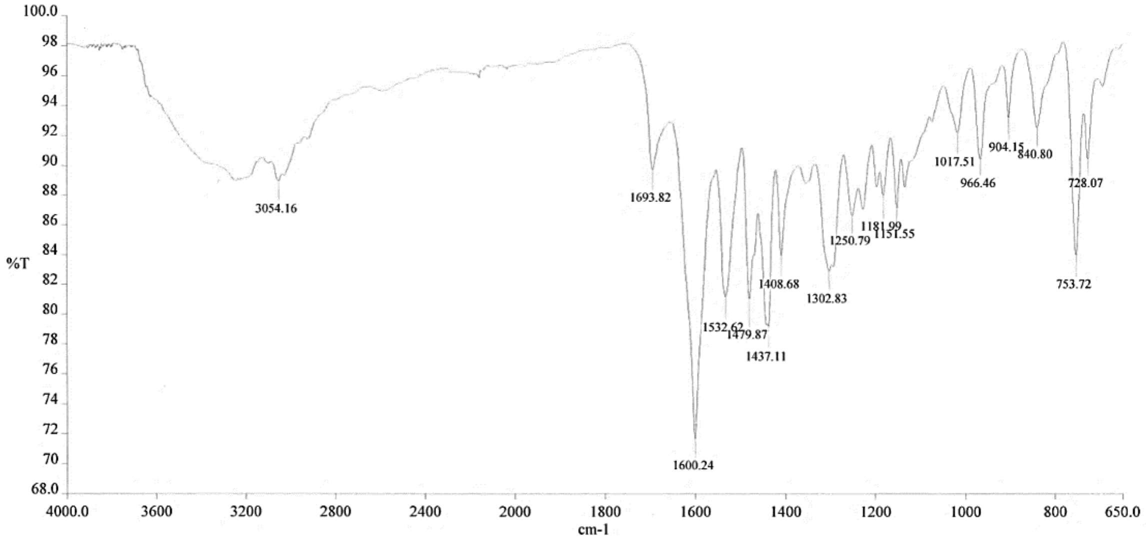

Figure S10. FTIR spectrum of compound **L2Pt**.

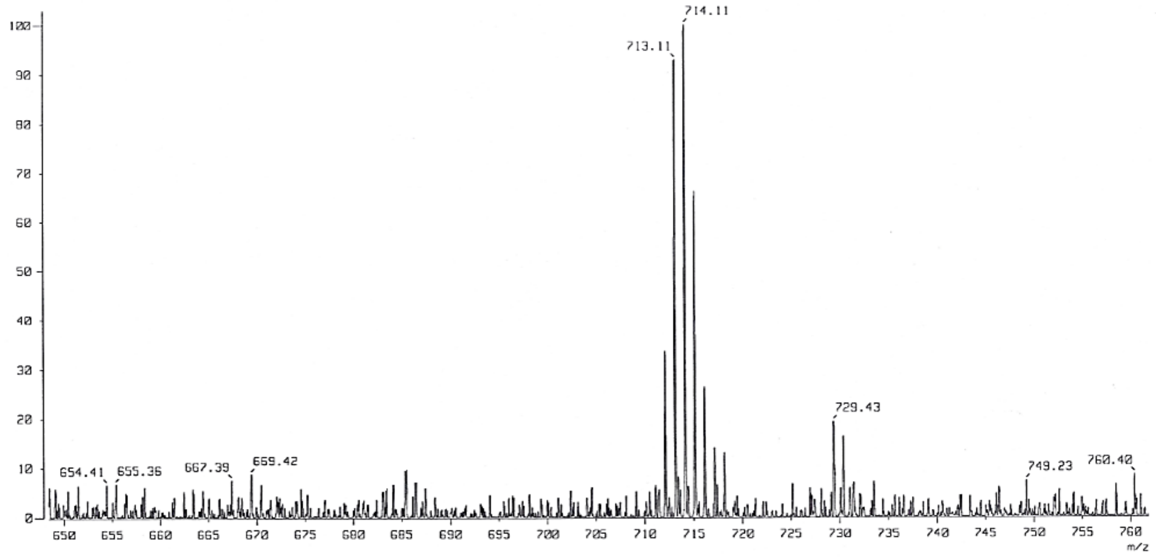

Figure S11. High resolution-FAB- mass spectrum of compound **L2Pt**.

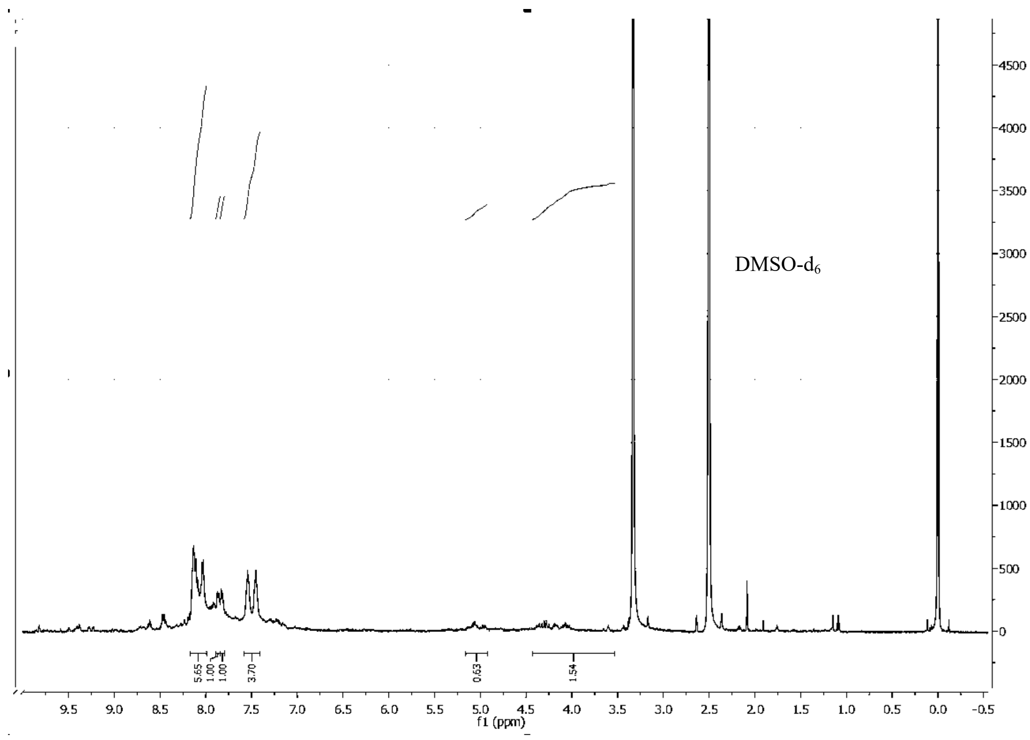

Figure S12. ^{1}H NMR spectrum of compound **L3Pt**.

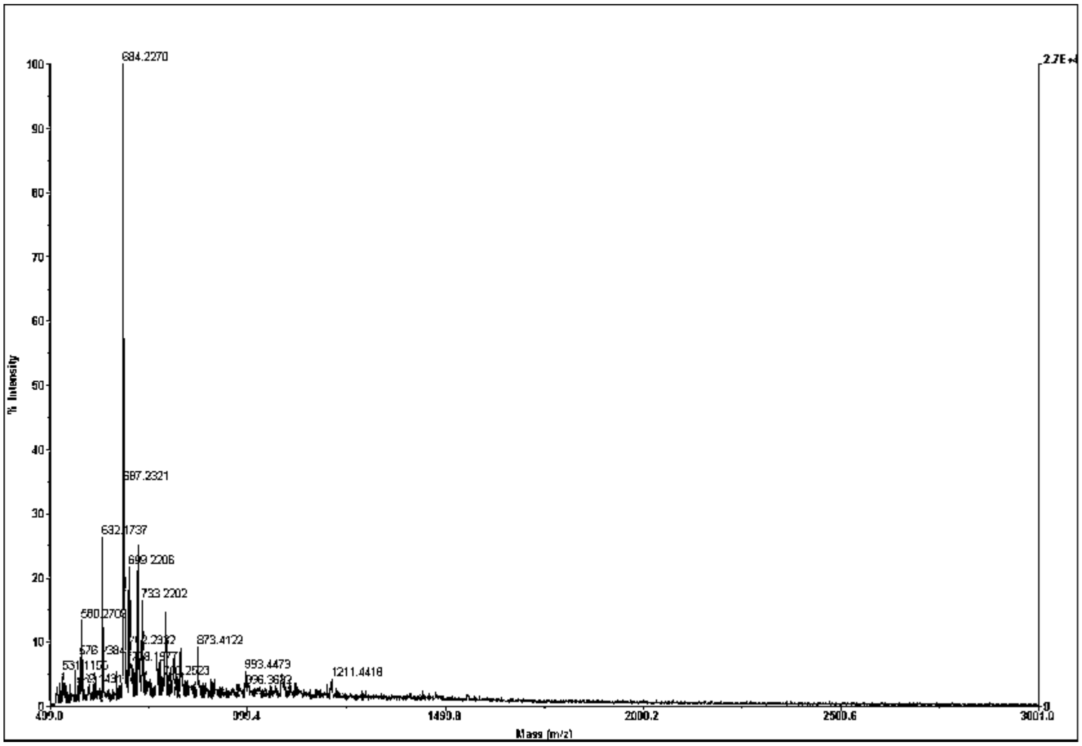

Figure S13. Maldi-tof mass spectrum of compound **L3Pt**.

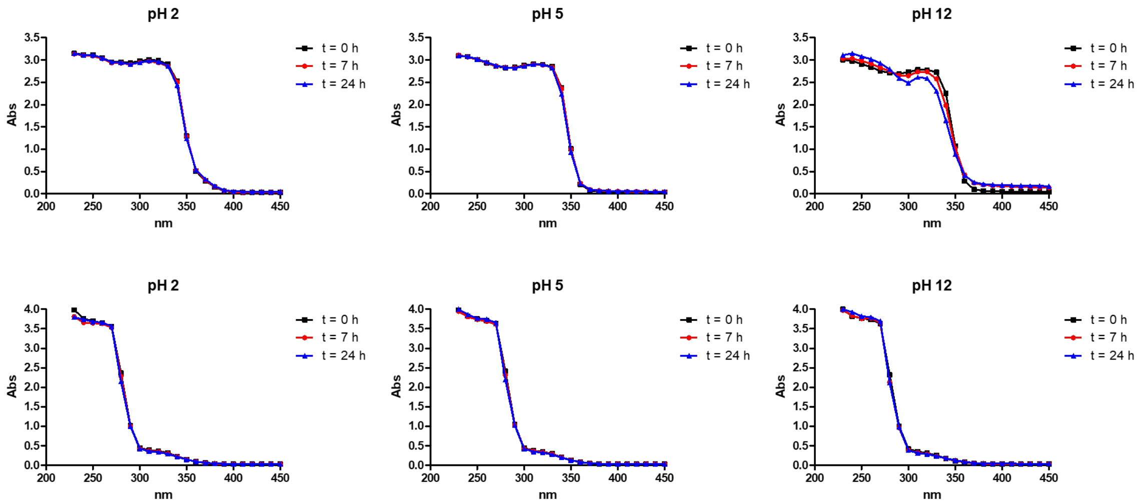

Figure S14. Time-dependent UV-Vis absorption spectra of compound **L1** (upper row) and **L1Pt** (lower row) in PBS solution recorded after t=0 h (black line), t=7 h (red line), and t=24 h (blue line) in different pH (e.g. strong acidic, weakly acidic, neutral, alkaline).

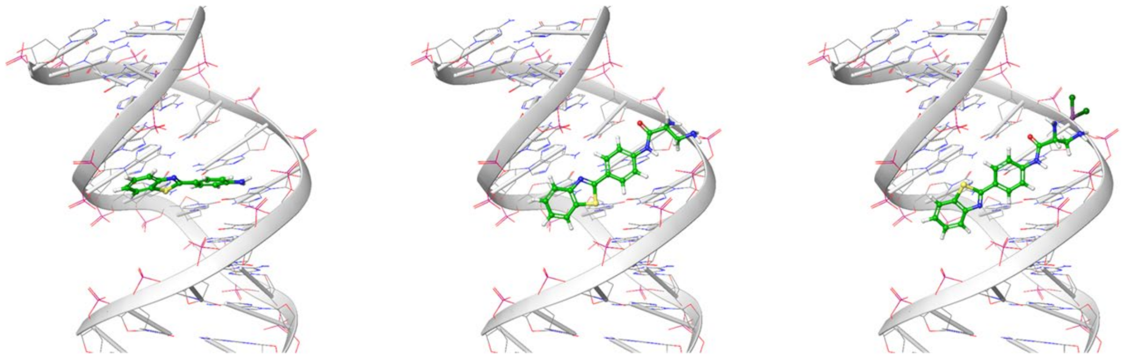

15 (A) 1LU5-BTA **15 (B) 1LU5-L1** **15 (C) 1LU5-L1Pt**

Figure S15. (A) Predicted binding pose of BTA on DNA (PDB ID: 1LU5). (B) Molecular docking simulation studies of the interaction between L1 and DNA (PDB ID: 1LU5). (C) Molecular docking simulation studies of the interaction between L1Pt and DNA (PDB ID: 1LU5).

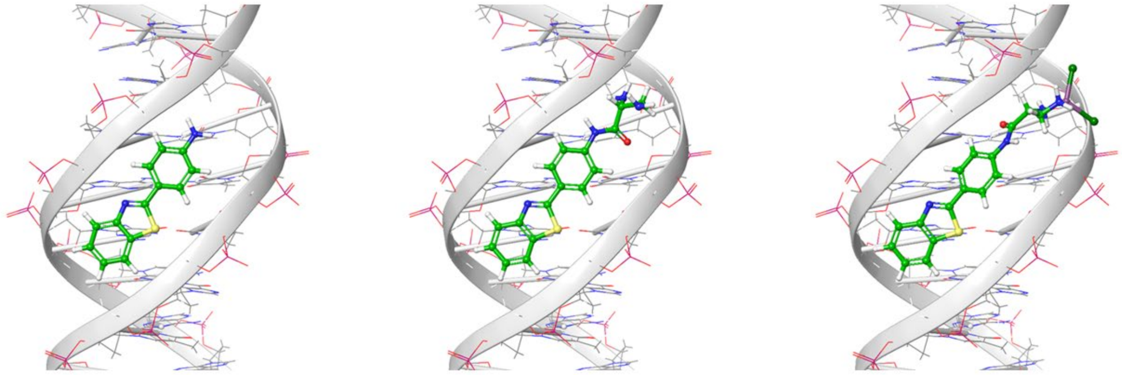

16 (A) 3CO3-BTA **16 (B) 3CO3-**L1 **16 (C) 3CO3-L1Pt**

Figure S16. (A) Predicted binding pose of BTA on DNA (PDB ID: 3CO3). (B) Molecular docking simulation studies of the interaction between L1 and DNA (PDB ID: 3CO3). (C) Molecular docking simulation studies of the interaction between L1Pt and DNA (PDB ID: 3CO3).
